# BK virus-associated hemorrhagic cystitis in pediatric stem cell transplantation: a case report and scoping review

**DOI:** 10.3389/fped.2023.1267678

**Published:** 2024-02-09

**Authors:** Julia E. Moss, William J. Muller

**Affiliations:** ^1^ Northwestern University Feinberg School of Medicine, Chicago, IL, United States; ^2^ Division of Infectious Diseases, Ann & Robert H. Lurie Children’s Hospital of Chicago, Chicago, IL, United States; ^3^ Department of Pediatrics, Northwestern University Feinberg School of Medicine, Chicago, IL, United States

**Keywords:** cystitis, stem cell transplantation, BK polyomavirus, viral infection, hematuria

## Abstract

**Introduction:**

BK virus-associated hemorrhagic cystitis (BK-HC) is a debilitating and poorly understood complication of hematopoietic stem cell transplantation (SCT). Hematuria, dysuria, and other symptoms associated with BK-HC are common in the immediate post-SCT period, making BK-HC difficult to distinguish from other conditions presenting with these symptoms. Despite published criteria for diagnosis, the degree to which these criteria are consistently applied to either clinical diagnosis or to studies informing BK-HC management is unclear. We present a case of BK-HC in a pediatric SCT recipient, and discuss the challenges associated with treatment in the absence of rigorous data to inform clinical management.

**Methods:**

We reviewed all cases of BK viruria at our center in patients undergoing SCT between January 2015 and December 2019. We then performed a scoping review of publications in PubMed addressing BK-HC, specifically focusing on how BK-HC was defined. Publications using the keywords “BK polyomavirus” and “hemorrhagic cystitis” were included if they involved a clinical study of SCT recipients and a full-text article was available in English. Case reports were excluded. Analysis focused on whether BK-HC was explicitly defined and whether the definition incorporated elements of diagnostic criteria published by European Conference on Infections in Leukemia (ECIL).

**Results:**

A total of 30 studies published between January 2018 and 30 June 2021 met criteria for review, including 4 clinical trials, 7 prospective observational studies, and 19 retrospective observational studies. Fifteen of these studies included pediatric patients (7 pediatric only, 8 combined adult and pediatric). Of the 30 publications, 19 included a definition of either BK-HC or BK cystitis, with only five using ECIL criteria, all of which were observational studies. Multiple interventions are described for treatment of BK-HC, including cidofovir, leflunomide, quinolones, hyperbaric oxygen, keratinocyte growth factor, and BK-specific cytotoxic T lymphocytes. However, evidence to support efficacy for any of these interventions is lacking.

**Discussion:**

Although BK-HC is a well-known complication of SCT, evidence to support available treatment options is limited. Well-controlled studies that incorporate clear diagnostic criteria are needed to better define the risk factors, natural history, and ideal interventions.

## 1 Introduction

BK polyomavirus (BKV) is a double-stranded DNA virus that causes persistent infection in the urinary tract (1). It is a ubiquitous virus, with up to 90% of individuals over age ten seropositive for BKV. While infection is largely asymptomatic in the general population, BKV contributes to morbidity in immunosuppressed populations including hematopoietic stem cell (SCT) recipients, with replication of the virus in the urinary tract contributing to BKV-associated hemorrhagic cystitis (BK-HC), an often debilitating and painful condition involving bleeding into the urinary bladder (2). Overlap between BK-HC and other causes of hematuria in the post-SCT setting pose challenges with diagnosis, making careful study of the condition to determine details of the epidemiology and pathophysiology difficult, and compromising our ability to identify effective interventions. Here we present a case of a patient with BK-HC, review the association of BK viruria and the diagnosis of BK-HC in our center's SCT population, discuss guidelines for BK-HC diagnosis, and review the literature to identify limitations of those guidelines and studies needed to better determine effective interventions.

## 2 Case description

The case presented here is a composite of more than one patient with similar presentation, modified to illustrate the challenges in diagnosis and management of BK-HC. Accordingly, informed consent was not applicable to this case description.

A 13-year-old female underwent haploidentical stem cell transplantation (SCT) for high-risk acute myelogenous leukemia (AML), and developed diffuse abdominal pain, right flank pain, dysuria, and hematuria on day 18 (D+18) following transplant.

The patient was previously healthy prior to her AML diagnosis. She presented about five months prior to SCT with fatigue, headache, several episodes of epistaxis, and swollen cervical lymph nodes. A complete blood count (CBC) at that time noted blasts, which led to an oncology referral and eventual AML diagnosis. Chemotherapy received in the weeks prior to her SCT admission included cytarabine, etoposide, daunorubicin, mitoxantrone, gemtuzumab, midostaurin, and fludarabine.

The patient's donor was her mother. Conditioning prior to SCT included busulfan, fludarabine, and thiotepa, with post-SCT cyclophosphamide given D+3. In the two weeks after transplant, she had intermittent nausea and epistaxis, and resolving mucositis. She started tacrolimus and mycophenolate mofetil for graft-vs-host disease (GVH) prophylaxis D+5.

Exam on D+18 was notable for discomfort related to abdominal pain, resolving oral erosions consistent with improving mucositis, and mild abdominal distention with diffuse pain upon palpation. CBC was notable for a white blood cell count of 900 cells/mcL, an absolute neutrophil count (ANC) of 645 cells/mcL, hemoglobin of 9.2 g/dL, and platelet count of 19,000/mcL. Urinalysis was notable for large blood, with microscopic examination reporting red blood cells too numerous to count.

Blood and urine were sent for bacterial stains and culture and for PCR testing for CMV, ADV, and BK virus. Ultrasonography of the kidneys and bladder was notable for non-specific debris within the bladder.

## 3 Differential diagnosis and diagnostic criteria for BK-HC

BK-HC develops between 2 and 8 weeks post-transplant in 8%–25% of pediatric SCT patients, and is associated with hematuria and urinary symptoms including frequency, urgency, and dysuria (3). Symptoms can persist for weeks to months and lead to severe complications such as bladder obstruction and acute kidney failure (3). While criteria for defining the grade of hemorrhagic cystitis of any cause are fairly well established in the literature (4), there is no specific biomarker for BK-HC, with BK viruria a necessary but not sufficient finding. Up to 80% of SCT patients shed BKV in the urine post-transplant, but only a minority develop urinary symptoms of BK-HC (5). The pathogenesis of BK-HC in SCT patients has not yet been fully elucidated (1), in part due to challenges with accurate diagnosis.

The differential diagnosis for post-SCT HC includes conditions which are fairly common in this patient population. Cyclophosphamide has been associated with HC, particularly that occurring within one week of myeloablative conditioning, though incidence may be ameliorated with mesna (6). Viruses other than BKV, including adenovirus and cytomegalovirus, are also potential causes of HC in the SCT population (7). Bacterial cystitis, bleeding disorders with or without thrombocytopenia, and mechanical complications from stents or catheters may also cause HC (3).

Consensus guidelines from the European Conference on Infections in Leukemia (ECIL) have proposed diagnostic criteria for BK-HC which include the “diagnostic triad” of: (1) clinical symptoms of cystitis (such as dysuria and lower abdominal pain), (2) hematuria grade 2 or higher (macroscopic), and (3) BK viruria >1 × 10^7^ copies/mL (3). These guidelines also note that BK-HC should be distinguished from early-onset HC occurring <1-week post-SCT, which may be due to the causes summarized above including adverse effects associated with conditioning.

## 4 Methods

### 4.1 Review of cases of BKV viruria in an SCT population

We reviewed all children at Ann & Robert H Lurie Children's Hospital of Chicago who underwent allogeneic SCT between January 2015 and December 2019 and were subsequently identified to have BKV viruria. This review was preliminary to a planned case-control analysis comparing patients with positive urine BK PCR who remained asymptomatic to those diagnosed with BK-HC. Patients were considered to have a clinical diagnosis of BK-HC if an attending physician specified this diagnosis in a progress note in the electronic medical record. The decision to send PCR testing for BK from urine or blood was at the discretion of the treating physician. The protocol was reviewed and approved by the local IRB (approval number IRB 2020-3324). This preliminary review identified several patients clinically diagnosed with BK-HC who did not fulfill ECIL diagnostic criteria (summarized further in Section 5.1).

### 4.2 Scoping review of BK-HC literature

The identified discrepancy between clinical BK-HC diagnosis and guideline recommendations prompted a scoping review of BK-HC literature. The scoping review methodology incorporates elements of narrative and systematic reviews (8, 9), combining a replicable literature search with a descriptive analysis of the literature generated, without formal assessment of the quality of the evidence reported in the identified manuscripts (8).

A PubMed search was conducted using the terms “BK polyomavirus” and “hemorrhagic cystitis” (all fields), filtered for studies published between 2018 and 2021 (i.e., after the introduction of the ECIL guidelines for BK-HC in January 2018). Articles covered in the review were required to meet the following inclusion criteria: (a) clinical study (e.g., clinical trial, prospective or retrospective observational study); (b) study population included hematopoietic stem cell transplant recipients (allogeneic and/or autologous); and (c) full article available online, in English. Case reports were excluded.

Articles were reviewed in detail and charted for core characteristics including study design, sample size, year of publication, and pediatric vs. adult population (vs. both). We recorded whether the article explicitly defined hemorrhagic cystitis (HC), BK virus-associated HC (BK-HC), and if BK-HC was defined, whether the definition incorporated each element of the ECIL diagnostic triad. Studies were reviewed to determine whether patients were excluded when HC onset was <1 week post-SCT or when an alternative etiology for HC was identified, whether papers directly stated that ECIL guidelines were utilized, and whether the ECIL guidelines were cited. For papers without clearly defined criteria for diagnosis of BK-HC in their study methods, we assessed BK-HC patient data provided in the results section (when available), to make inferences about the diagnostic criteria utilized. This part of the analysis intended to identify factors considered important in diagnosis across institutions that may not be part of ECIL guidelines.

## 5 Results

### 5.1 Analysis of SCT patients with BK viruria

We identified 43 children at our center with a history of BK viruria following allogeneic SCT (Supplementary Table S1). There were a total of 151 allogeneic SCT in 149 patients over the time period studied. Of those, 26 patients had a documented clinical diagnosis of BK-HC in the medical record, five of whom (19.2%) met only one or two of the three ECIL diagnostic criteria. Two patients had urine BK PCR copy numbers below the ECIL threshold of 10^7^ copies per mL, two had microscopic hematuria only (grade 1), and one had no documented clinical symptoms. Additionally, one of the patients with urine BK PCR copy number below 10^7^ copies/mL had concomitant adenovirus cystitis, further complicating the diagnosis of BK-HC. Two additional patients with a clinical diagnosis of BK-HC had onset of disease prior to D+7 (i.e., “early onset” HC), which ECIL guidelines categorize as too early for BK-HC and more likely due to toxicity of the conditioning regimen; both had total body irradiation as part of pre-transplant conditioning, and one also had cyclophosphamide treatment. Therefore, as many as 7 of 26 patients with a clinical diagnosis of BK-HC (26.9%) would not have clearly been categorized as BK-HC by a strict reading of ECIL guidelines.

Of the remaining 17 SCT patients with BK viruria but no clinical diagnosis of BK-HC, it is possible that at least two (11.8%) may have satisfied the ECIL diagnostic triad with additional clinical data. One patient with urinary urgency and macroscopic hematuria with clots had one reported urine BK PCR of 7 × 10^6 ^copies/mL, but the urine was not checked again for BK. One patient with grade 2 hematuria and 1 × 10^9^ copies/mL BK in urine, but no reported symptoms of BK-HC, was receiving pain control for mucositis which could plausibly have masked abdominal pain and/or complicated assessment of urinary retention.

Although the single center nature of this analysis is limited by patient numbers, this review of patients at our center illustrates two related and non-exclusive sets of challenges: (a) clinical diagnosis of BK-HC may be overly permissive in at least some patients, and (b) existing guideline criteria may be overly restrictive in some patients, excluding some thought clinically to have a diagnosis of BK-HC.

### 5.2 Scoping literature review

We reviewed literature published after publication of the ECIL guidelines to determine whether (a) these guidelines were incorporated into the study definition for BK-HC, (b) if they were not, how BK-HC was defined, and (c) whether our observations that clinical diagnosis of BK-HC at our center did not consistently align with ECIL guideline criteria for the diagnosis were also a characteristic of the literature.

#### 5.2.1 Characteristics of identified manuscripts

The initial literature search generated 78 results, including 33 clinical studies, 17 case reports, and 28 non-clinical studies (e.g., translational or basic science, systematic reviews, meta-analyses, narrative reviews, guideline summaries). Among the 33 studies eligible based on study type (clinical studies which were not case reports), one did not include SCT patients (the focus was HIV patients) (10), one was not available in English (11), and one did not have full text availability (12). Thirty studies were ultimately included in the analysis.

Among the 30 included studies, 4 were clinical trials, 7 were prospective observational studies, and 19 were retrospective observational studies. 27 studies included allogeneic hematopoietic stem cell transplant patients only, one study included both allogeneic and autologous SCT patients (13), one study included both SCT and solid organ transplant patients (14), and one study did not specify SCT type (15). Fifteen of the 30 studies included pediatric patients (7 studies pediatric only, 8 combined adult and pediatric population).

#### 5.2.2 Studies applying ECIL criteria to diagnosis of BK-HC

Five of the 30 studies analyzed (16.7%) defined BK-HC using the ECIL diagnostic triad (16–20). This included two prospective and three retrospective studies, with between 15 and 67 patients with BK-HC included in each study (Table 1). Only one of the five studies included pediatric patients. There was no obvious correlation between year of study publication and application of ECIL criteria.

**Table 1:** Scoping review of BK-HC literature.

Reference	Study design	Age group	Number of SCT patients in study (total)	Number of patients with BK-HC	BK-HC definition (where different from ECIL)
Studies which applied ECIL criteria					
Arango (16)	Prospective	Adult	85	15	ECIL criteria used
Kaphan (17)	Retrospective	Adult	157	43	ECIL criteria used
Kerbauy (18)	Retrospective	Adult	133	36	ECIL criteria used
Oltolini (19)	Prospective	Adult	235	36	ECIL criteria used
Ruderfer (20)	Retrospective	Pediatric	314	67	ECIL criteria used
Studies which did not clearly specify BK-HC criteria					
Foster (21)	Retrospective (treatment)	Pediatric	10	10	Clinical BK-HC diagnosis and patient received intravesicular cidofovir treatment directed at BK-HC
McGuirk (22)	Retrospective	Adult and pediatric	13,363	343^a^	Clinical diagnosis based on diagnosis code
Gander (15)	Retrospective	Pediatric	39	34	Presumably any positive BK PCR in urine
Gutierrez-Aguirre (23)	Retrospective	Adult and pediatric	111	10	Applied established criteria for HC; presumably used any positive BK PCR (blood or urine) for BK-HC
Nelson (14)	Clinical trial	Adult and pediatric	38	38	Presumably any positive BK PCR in urine or >1 × 10^3^ copies/mL in plasma
Mustafa (24)	Retrospective	Pediatric	34	1	Any positive BK PCR in urine; patient was also positive for CMV in urine
Parta (25)	Clinical trial	Adult and pediatric	7	4	Presumably any grade hematuria and any positive BK PCR (blood or urine)
Umeda (26)	Retrospective	Pediatric	112	10	Applied established criteria for HC; presumably used any positive BK PCR (blood or urine) for BK-HC
Castagna (27)	Clinical trial	Adult	23	1	Not defined; presumably used clinical diagnosis
Chang (28)	Retrospective	Adult	187	29	Not defined; presumably used any positive BK PCR (blood or urine)
Karantanos (29)	Prospective	Adult	27	9	Not defined; presumably any grade hematuria and any positive BK PCR (blood or urine)
Studies which specified BK-HC criteria which differed from ECIL					
Atilla (30)	Prospective	Adult	59	18	Any positive BK PCR in plasma
Coomes (31)	Retrospective (treatment)	Adult	12	12	Any grade hematuria and any positive BK PCR (blood or urine)
Hosokawa (32)	Retrospective (treatment)	Adult	16	12	Any positive BK PCR in urine
Imlay 2020 (33)	Retrospective	Adult	128	128	Positive BK PCR in both blood and urine
Jaiswal (34)	Prospective	Adult and pediatric	115	6	Any positive BK PCR in urine; did not specify whether symptoms were required
Kesherwani (35)	Retrospective	Adult	96	8	Any positive BK PCR in urine
Laskin (36)	Prospective	Adult and pediatric	193	43	Kidney-related outcomes including HC were assessed, with BK PCR > 1 × 10^9^ copies/mL in urine found to be one risk factor for HC. Clinical diagnosis of BK-HC was not a primary focus of the study, which cited ECIL guidelines.
Masieri (37)	Retrospective (treatment)	Adult	10	10	Positive BK PCR in both blood and urine, urine BK PCR > 9 × 10^6^ copies/mL
Mayer (38)	Retrospective (treatment)	Adult	166	22	Any positive BK PCR in urine
Olson (39)	Clinical trial	Adult and pediatric	59	59	Any positive BK PCR in urine
Onda (40)	Retrospective	Adult and pediatric	207	39	Any grade hematuria and any positive BK PCR in urine
Salamonowicz-Bodzioch (41)	Prospective	Pediatric	133	36	Any grade hematuria and any positive BK PCR (blood or urine), and treatment directed at BK-HC provided
Tong (42)	Retrospective (treatment)	Pediatric	13	13	Urine BK PCR > 1 × 10^6^ copies/mL
Tooker (13)	Retrospective (treatment)	Adult	33	33	Any positive BK PCR in urine and intravesicular cidofovir treatment directed at BK-HC provided

^a^ Unable to distinguish BK from EBV or JC based on diagnosis code.

Among the five studies which applied the ECIL diagnostic triad, there was variation in whether timing of BK-HC onset or exclusion of alternative HC etiologies was accounted for in the inclusion of patients in the analysis. Only two of the five studies explicitly mentioned exclusion of alternative HC etiologies (18, 20), while one study noted that patients with concurrent bacterial cystitis were not excluded from the case population (17). None of the five studies specified timeframe requirements for presentation of BK-HC. One study reported a range of 2–372 days post-SCT for BK-HC onset (as part of the results), suggesting that the ECIL recommendation to exclude patients presenting with HC < 1-week post-SCT was not applied (18).

#### 5.2.3 Application of diagnostic definitions of BK-HC differing from ECIL criteria

Eleven of the 30 studies analyzed (36.7%) reported on BK-HC without clearly specifying the criteria used in the Methods (Table 1), including two retrospective studies which used clinically diagnosed BK-HC for inclusion (21, 22), six studies in which criteria needed to be inferred by review of the Results (14, 15, 23–26), and three studies in which criteria remained unclear after review of the Results (27–29). Eight of the 11 studies in this category included pediatric patients, including four which were exclusively in children. Two were clinical trials, one of which involved treatment with virus-specific T cells for BK-associated disease (14), and the other assessing outcomes (including BK-HC) of a conditioning regimen using post-SCT cyclophosphamide in patients with a primary immune deficiency (25). Five additional studies were assessing outcomes of either treatment directed at BK-HC (21, 27) or of outcomes of different SCT regimens (23, 24, 26).

The remaining 14 studies analyzed (46.7%) used diagnostic definitions of BK-HC which differed from the ECIL triad (Table 1) (13, 30–42). Six of these studies included pediatric patients, including two which were exclusively in children. These studies generally used less restrictive criteria than ECIL, most commonly allowing any positive BK PCR result for a case to be considered related to the virus. Two studies applied criteria for diagnosis that were more restrictive than the ECIL triad, both of which required both blood and urine to be PCR positive for BK virus (33, 37). The Imlay et al. study additionally required BK-HC cases to present with HC after either platelet engraftment or D+28 (whichever was earlier) for inclusion, a timeframe noted by the authors to be stricter than the ECIL requirement of >1-week post-SCT (33). Laskin et al. analyzed prospectively collected samples to identify associations between kidney-related outcomes and BK virus replication in SCT recipients, reporting that a threshold for PCR copy-number of >10^9^ copies/mL was predictive of HC, though this group was mindful of the ECIL criteria in their definition (36). One clinical trial was included among the studies which used criteria that differed from the ECIL triad (39). This study assessed treatment with virus-specific T-cells for BK-HC and included patients with any positive BK PCR test of the urine.

## 6 Patient outcome

**Figure 1:**
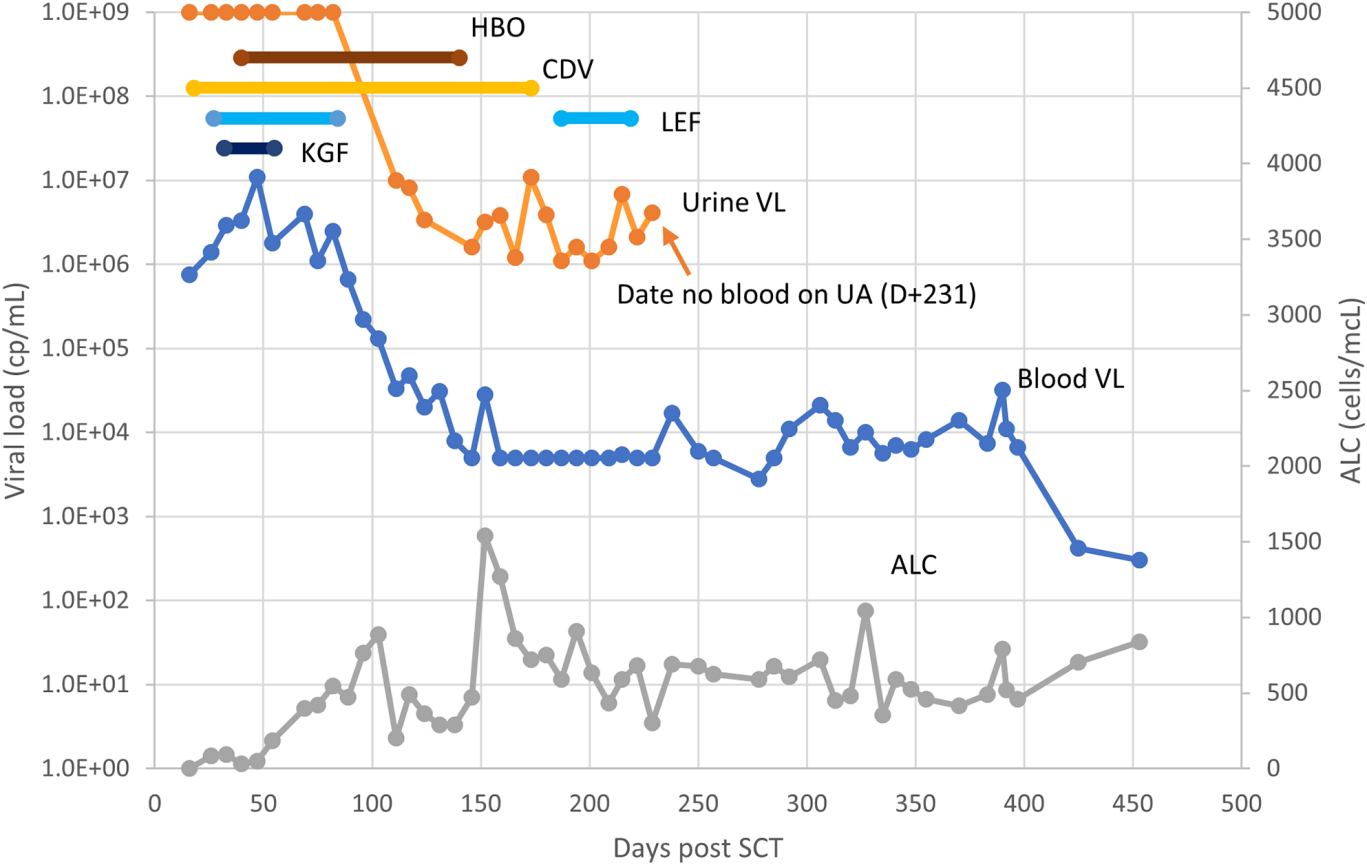
Clinical course of BK-HC in reported case. Symptoms for the patient continued between D+18 and D+250, primarily consisting of dysuria and hematuria. Interventions attempted included hyperbaric oxygen (HBO), cidofovir (CDV), leflunomide (LEF), and keratinocyte growth factor (KGF). Other abbreviations- VL, viral load (PCR copy number); ALC, absolute lymphocyte count.

For the composite patient presented in Section 2, PCR testing of the urine on D+18 was positive at more than 1 × 10^9^ copies/mL; this patient therefore satisfied the ECIL criteria for BK-HC. Intravenous cidofovir was started. Symptoms persisted for several months, including dysuria, intravesicular clots, and abdominal pain, along with persistently positive testing of urine and blood by PCR for BK (Figure 1). Because of both the concerning clinical course and the lack of clearly established interventions, in addition to the prolonged course of cidofovir (43) there were a number of reported treatment options pursued, including leflunomide (44, 45), keratinocyte growth factor (46, 47) and hyperbaric oxygen (48). There was no clear response to any of these interventions; instead, resolution of clinical symptoms, hematuria, and positive BK testing in blood and urine more clearly correlated with sustained improvement in absolute lymphocyte count.

## 7 Discussion

Clinical diagnosis of BK-HC after stem cell transplantation is challenging. Factors which affect risk for hemorrhagic cystitis include toxicity of the conditioning regimen, risk of cystitis from a number of bacteria and viruses related to low leukocyte counts and immunosuppressive medications implemented to minimize risk of GVH, and low platelet counts associated with the transplant regimen. HC related to conditioning is thought to be specifically promoted by busulfan, cyclophosphamide, and total body irradiation, and is associated with earlier onset, often occurring within a week of cell infusion (3). Cyclophosphamide dosing after SCT cell infusion as part of GVH prophylaxis may also promote HC, including HC attributed to BK replication (3). The immunologic capacity of the patient to respond to viral replication may also influence the natural history of BK-HC, as suggested by our patient's resolution of disease in association with improving ALC and by studies suggesting responses to BK-specific CTLs as treatment (14, 39).

The 2018 ECIL publication of criteria for diagnosis of BK-HC was part of an effort to provide evidence-based recommendations for preventing, diagnosing, and treating BK-HC (3). Our analysis of the literature published since the release of the ECIL criteria suggests substantial variability in the criteria applied to diagnosis of BK-HC for clinical and/or research investigations, with less than 20% of publications applying the ECIL criteria and more than 35% failing to clearly provide criteria used for diagnosis. This is despite the observation that 14 of the 30 analyzed studies cited the ECIL guidelines. The lack of consistent application in different studies can lead to challenges in interpretation of reported risk factors for disease, and makes evaluation of possible efficacy for potential treatment options problematic. Indeed, several of the possible treatments that have been put forward for this condition were attempted in the case patient despite lack of evidence to support efficacy; contributors to this lack of evidence include the variability in diagnostic criteria applied by different study teams and the often retrospective nature of the studies reporting these interventions. This challenge in interpreting evidence for different BK-HC treatment options is magnified in the pediatric setting, in which there are even less data, and highlights the possible alternatives that the ECIL guidelines are not sufficiently broad for clinical use or that clinical diagnosis of BK-HC is overly broad. Both possibilities are reflected in our review of our center's data (Section 5.1 and Supplementary Table S1). Notably, several of our center's patients were diagnosed prior to publication of ECIL criteria.

It is worth noting that our analysis of the literature may have been limited to a certain extent by the search strategy used. Several of the publications analyzed intended to identify risk factors for BK-HC or other complications of SCT in their population, including threshold levels of DNA copy number in urine or plasma, which preclude application of the ECIL criteria for DNA copy number as that was one of the study variables. Nevertheless, several of the analyzed studies were clinical trials or reviews of outcomes of treatment options for BK-HC, some of which used a clinical diagnosis of BK-HC as sufficient. Although it may be argued that criteria (including the ECIL criteria) which do not capture the clinical population affected may not be sufficient for study of the disease process, the lack of application of a consistent and clear definition of the condition makes comparative interpretation of many studies challenging. Discrepancies between what clinicians are treating as BK-HC and what patients clearly satisfy guideline criteria for diagnosis could suggest that better definitions may be needed, or that additional biomarkers might need to be evaluated. Specifically, BK viruria or plasma DNAemia may not be sufficiently sensitive or specific. It is additionally possible that more widespread education on the ECIL criteria is needed, so that clinicans more consistently apply them to BK-HC diagnosis. We acknowledge also that our review of our own center's experience is limited by potential inaccuracies in documentation.

## 8 Recommendations for BK-HC and future directions for investigation

Management of infectious complications in immune-compromised patients has become a specialty of its own, largely due to the complexity of the patient population and rapidly changing interventions being implemented and studied. Although BK-HC is not a newly recognized complication of SCT (and less commonly SOT), a detailed understanding of pathogenesis and treatment remains somewhat elusive. Specific recommendations and directions for future efforts, informed by our analysis of recent literature, include:

- Collaborative efforts to retrospectively and/or prospectively review the natural history of hemorrhagic cystitis after defined conditions of SCT, particularly in children, would be of great benefit. Prospective studies could help address the limitations of retrospective studies (such as unclear documentation in the medical record and challenges in distinguishing BK-HC from other forms of HC), as we saw with our review.
- Similarly, prospective observational studies could identify criteria to better predict risk factors for BK-HC (and other forms of HC), and determine criteria to be used to assess response to different interventions.
- Interventional studies for possible BK-HC treatments need clearly stated inclusion criteria and endpoints, and consistently applied diagnostic criteria are critical to interpretation. Researchers, sponsors, and reviewers need to collaborate to ensure that consistently applied diagnostic criteria are used.
- Additional biomarkers that indicate when BK or other causes are the primary driver of HC could inform future work. Although data which clearly show that host immunity leads to resolution of BK-HC are lacking, studies suggesting benefit after BK virus-specific T-cell infusions support the idea that immunologic evaluations of host response such as BK-specific T-cell responses might be one relevant biomarker (14, 39).
- Similarly, identification of biomarkers that predict risk of developing BK-HC, prognosis and/or duration of BK-HC, or which allow monitoring response to therapy would be of benefit to the community.

## Data Availability

The original contributions presented in the study are included in the article/Supplementary Material, further inquiries can be directed to the corresponding author.
