# Supplementary material for: BK virus-associated hemorrhagic cystitis in pediatric stem cell transplantation: a case report and scoping review
Source: Front Pediatr. 2024 Feb 9;11:1267678. doi: 10.3389/fped.2023.1267678 (PMC10884191; doi:10.3389/fped.2023.1267678)
Supplement: Supplementary file 1 [file Table1.pdf]

Supplementary Table 1. Characteristics of SCT patients with positive PCR testing for BK in urine

Sex	Age at transplant (years)	Underlying diagnosis	Conditioning regimen	Days from SCT to BK positive urine >1×10^7 copies/mL	ECIL criteria satisfied?	Clinical diagnosis BK-HC?
M	22	Evan's syndrome	ATG, Busulfan, Fludarabine, Thiotepa	31	Y	Y
F	19	B-ALL	ATG, Cyclophosphamide, Thiotepa, TBI	29	Y	Y
M	16	Ph-positive ALL	ATG, Cyclophosphamide, Thiotepa, TBI	49	Y	Y
M	17	AML	ATG, Busulfan, Fludarabine, TBI	93	Y	Y
M	16	B-ALL	Fludarabine, Thiotepa, TBI	34	Y	Y
F	12	AML	Busulfan, Fludarabine, TBI	53	Y	Y
F	18	AML	ATG, Cyclophosphamide, Thiotepa, TBI	14	Y	Y
M	5	AML	ATG, Busulfan, Fludarabine, TBI	46	Y	Y
M	12	B-ALL	Cyclophosphamide, Etoposide, TBI	23	Y	Y
M	11	Aplastic anemia	ATG, Fludarabine, Cyclophosphamide, TBI	26	Y	Y
M	10	Ph-positive ALL	Cyclophosphamide, Etoposide, TBI	14	Y	Y
M	18	B-ALL	ATG, Busulfan, Fludarabine, TBI	41	Y	Y
M	15	AML	ATG, Busulfan, Cyclophosphamide, Thiotepa	55	Y	Y
M	10	AML	ATG, Busulfan, Cyclophosphamide, Thiotepa	17	Y	Y
F	12	AML	Busulfan, Fludarabine, Thiotepa	20	Y	Y
F	15	AML	Busulfan, Fludarabine, Thiotepa	16	Y	Y
F	13	AML	ATG, Fludarabine, Busulfan, TBI	22	Y	Y
F	22	Aplastic anemia	ATG, Fludarabine, Cyclophosphamide, TBI	33	Y	Y
M	18	B-ALL	ATG, Cyclophosphamide, Thiotepa, TBI	7	Y	Y
M	13	T-ALL	Cyclophosphamide, Etoposide, TBI	143	Peak VL <10^7, concomitant ADV in urine	Y
F	13	MDS/RAEBT*	Busulfan, Fludarabine, TBI	168	Peak VL <10^7	Y
F	7	Primary immune deficiency	Busulfan, Fludarabine, Cyclophosphamide, TBI	32	Microscopic hematuria only	Y
M	4	Metachromatic leukodystrophy	ATG, Fludarabine, Busulfan	88	Microscopic hematuria only	Y
M	15	Ph-positive ALL	ATG, Cyclophosphamide, Thiotepa, TBI	43	No clinical symptoms	Y
M	21	B-ALL	ATG, Cyclophosphamide, Thiotepa, TBI	5	Before D+7	Y

M	16	T-cell lymphoma	Fludarabine, Thiotepa, TBI	6	Before D+7	Y
F	16	Ph-positive ALL	Cyclophosphamide, Etoposide, TBI	9	N	N
F	22	B-ALL	ATG, Cyclophosphamide, Thiotepa, TBI	13	N	N
M	8	B-ALL	Cyclophosphamide, Etoposide, TBI	62	N	N
F	13	B-ALL	Cyclophosphamide, Etoposide, TBI	14	N	N
M	6	B-ALL	Cyclophosphamide, Etoposide, TBI	54	N	N
F	17	B-ALL	ATG, Fludarabine, Cyclophosphamide	171	N	N
M	13	B-ALL	Cyclophosphamide, Etoposide, TBI	580	N	N
F	11	T-ALL	ATG, Cyclophosphamide, Thiotepa, TBI	10	N	N
M	10	Aplastic anemia	ATG, Fludarabine, Cyclophosphamide	34	N	N
F	11	Aplastic anemia	ATG, Fludarabine, Cyclophosphamide	65	N	N
M	3	AML	ATG, Busulfan, Fludarabine	45	N	N
M	18	B-ALL	ATG, Cyclophosphamide, Thiotepa, TBI	38	N	N
F	14	Aplastic anemia	ATG, Fludarabine, Cyclophosphamide	1	N	N
F	13	Thalassemia major	Alemtuzumab, Fludarabine, Melphalan, Thiotepa	16	N	N
F	16	AML	Busulfan, Fludarabine, Thiotepa	6	N	N
F	20	T-ALL	Cyclophosphamide, Thiotepa, TBI	9	N	N
F	12	Biphenotypic leukemia	Cyclophosphamide, Thiotepa, TBI	18	N	N

*MDS/RAEBT: Myelodysplastic syndrome/ Refractory Anemia with Excess Blasts in Transformation
